# Supplementary material for: Evaluation of a point-of-care rapid diagnostic test kit (SICKLECHECK) for screening of sickle cell diseases
Source: PLoS One. 2024 Aug 16;19(8):e0309045. doi: 10.1371/journal.pone.0309045 (PMC11329129; doi:10.1371/journal.pone.0309045)
Supplement: Attachment — Submitted filename: PONE-D-24-04629.pdf [file pone.0309045.s003.pdf]

PLOS ONE

Evaluation of a point-of-care rapid diagnostic test kit (SICKLECHECKTM) for screening of sickle cell disorders

--Manuscript Draft--

**Manuscript Number:**	PONE-D-24-04629
**Article Type:**	Research Article
**Full Title:**	Evaluation of a point-of-care rapid diagnostic test kit (SICKLECHECKTM) for screening of sickle cell disorders
**Short Title:**	Point-of-care rapid diagnostic test kit for screening of sickle cell disorders
**Corresponding Author:**	Samira Kumar Behera, M.D.<br>Maharaja Krishna Chandra Gajapati Medical College and Hospital<br>Berhampur, Odisha INDIA
**Keywords:**	Sickle Cell Disorder; SICKLECHECKTM; Rapid Diagnostic Test; Early Diagnosis
**Abstract:**	Sickle cell disorders (SCD) are the most common genetic disorders with significant morbidity and mortality worldwide, including in India. The high prevalence of this disorder in many geographical regions calls for the use of a point-of-care rapid diagnostic test (RDT) for early screening and management of the diagnosed cases to reduce the allied clinical severity. In view of this, the present study was undertaken for the validation of a point-of-care RDT kit (SICKLECHECKTM) for the screening of SCD. This validation and diagnostic accuracy study was conducted among the cases advised for screening of SCD. For validation, all the recruited cases were investigated for both the SICKLECHECKTM RDT kit and HPLC (Variant-II) considering HPLC as a gold standard. A total of 400 cases were screened for both tests. For the presence and absence of sickle cell hemoglobin in the samples, SICKLECHECKTM RDT kit results showed a sensitivity and specificity of 99.39% and 98.73% respectively with references to HPLC findings. For the detection of the 'AS' pattern, the SICKLECHECKTM RDT kit has shown a sensitivity and specificity of 99.07% and 98.81% respectively. For the detection of the 'SS' pattern, the SICKLECHECKTM RDT kit has shown a sensitivity and specificity of 97.92% and 100.0% respectively. Cases with β thalassemia trait, hemoglobin E trait, hemoglobin Lepore trait and trait for hereditary-persistence-of-fetal-hemoglobin (high HbF %) diagnosed in HPLC were resulted with 'AA' pattern in SICKLECHECKTM RDT kit. The high sensitivity and specificity of the SICKLECHECKTM RDT kit insist on its use as a point-of-care screening tool for SCD especially where there is a lack of laboratory facilities as well as in hospital-based set-up requiring immediate diagnosis and management of SCD. However, for further confirmation, the samples should be analyzed with other gold standard techniques like HPLC.
**Order of Authors:**	Prasanta Purohit
	Chinmayee Parida
	Tapan Kumar Martha
	Snahal Bholo
	Aparupa Naik
	Samira Kumar Behera
**Additional Information:**	
**Question**	**Response**
**Financial Disclosure**<br><br>Enter a financial disclosure statement that describes the sources of funding for the work included in this submission. Review the submission guidelines for detailed	Yes

Powered by Editorial Manager® and ProduXion Manager® from Aries Systems Corporation

requirements. View published research articles from *PLOS ONE* for specific examples.<br><br>This statement is required for submission and **will appear in the published article** if the submission is accepted. Please make sure it is accurate.<br><br>**Funded studies**<br>Enter a statement with the following details:<br>• Initials of the authors who received each award<br>• Grant numbers awarded to each author<br>• The full name of each funder<br>• URL of each funder website<br>• Did the sponsors or funders play any role in the study design, data collection and analysis, decision to publish, or preparation of the manuscript?<br><br>Did you receive funding for this work?	
Please add funding details.<br>as follow-up to "**Financial Disclosure**<br><br>Enter a financial disclosure statement that describes the sources of funding for the work included in this submission. Review the submission guidelines for detailed requirements. View published research articles from *PLOS ONE* for specific examples.<br><br>This statement is required for submission and **will appear in the published article** if the submission is accepted. Please make sure it is accurate.	This study was funded by Department of Health Research, Ministry of Health and Family Welfare, Government of India (Letter No. V.25011/570(i)/2010-HR Dt.07.01.2014) for the establishment of a Multi-Disciplinary Research Unit (MRU) at Maharaja Krishna Chandra Gajapati Medical College, Berhampur, Odisha, India.

Powered by Editorial Manager® and ProduXion Manager® from Aries Systems Corporation

**Funded studies**<br>Enter a statement with the following details:<br>• Initials of the authors who received each award<br>• Grant numbers awarded to each author<br>• The full name of each funder<br>• URL of each funder website<br>• Did the sponsors or funders play any role in the study design, data collection and analysis, decision to publish, or preparation of the manuscript?<br><br>Did you receive funding for this work?"	
Please select the country of your main research funder (please select carefully as in some cases this is used in fee calculation).<br>as follow-up to "**Financial Disclosure**<br><br>Enter a financial disclosure statement that describes the sources of funding for the work included in this submission. Review the submission guidelines for detailed requirements. View published research articles from *PLOS ONE* for specific examples.<br><br>This statement is required for submission and **will appear in the published article** if the submission is accepted. Please make sure it is accurate.<br><br>**Funded studies**<br>Enter a statement with the following details:<br>• Initials of the authors who received each award<br>• Grant numbers awarded to each author<br>• The full name of each funder<br>• URL of each funder website<br>• Did the sponsors or funders play any role in the study design, data collection and analysis, decision to publish, or preparation of the manuscript?<br><br>Did you receive funding for this work?"	INDIA - IN
**Competing Interests**	The authors have declared that no competing interests exist.

Use the instructions below to enter a competing interest statement for this submission. On behalf of all authors, disclose any competing interests that could be perceived to bias this work—acknowledging all financial support and any other relevant financial or non-financial competing interests.<br><br>This statement is required for submission and **will appear in the published article** if the submission is accepted. Please make sure it is accurate and that any funding sources listed in your Funding Information later in the submission form are also declared in your Financial Disclosure statement.<br><br>View published research articles from *PLOS ONE* for specific examples.<br><br>**NO authors have competing interests**<br><br>Enter: *The authors have declared that no competing interests exist.*<br><br>**Authors with competing interests**<br><br>Enter competing interest details beginning with this statement:<br><br>*I have read the journal's policy and the authors of this manuscript have the following competing interests: [insert competing interests here]*<br><br>* typeset	
**Ethics Statement**<br><br>Enter an ethics statement for this submission. This statement is required if the study involved:<br><br>• Human participants<br>• Human specimens or tissue<br>• Vertebrate animals or cephalopods<br>• Vertebrate embryos or tissues	This study was approved by the Institutional Ethical Committee of M.K.C.G Medical College, Berhampur, Odisha (Ref No 1298/Chairman-IEC, M.K.C.G. Medical College, Brahmapur-4, Date 05. 04.2023)

• Field research<br><br>Write "N/A" if the submission does not require an ethics statement.<br><br>General guidance is provided below. Consult the submission guidelines for detailed instructions. **Make sure that all information entered here is included in the Methods section of the manuscript.**	

Powered by Editorial Manager® and ProduXion Manager® from Aries Systems Corporation

**Format for specific study types**<br><br>**Human Subject Research (involving human participants and/or tissue)**<br>• Give the name of the institutional review board or ethics committee that approved the study<br>• Include the approval number and/or a statement indicating approval of this research<br>• Indicate the form of consent obtained (written/oral) or the reason that consent was not obtained (e.g. the data were analyzed anonymously)<br><br>**Animal Research (involving vertebrate animals, embryos or tissues)**<br>• Provide the name of the Institutional Animal Care and Use Committee (IACUC) or other relevant ethics board that reviewed the study protocol, and indicate whether they approved this research or granted a formal waiver of ethical approval<br>• Include an approval number if one was obtained<br>• If the study involved *non-human primates*, add *additional details* about animal welfare and steps taken to ameliorate suffering<br>• If anesthesia, euthanasia, or any kind of animal sacrifice is part of the study, include briefly which substances and/or methods were applied<br><br>**Field Research**<br><br>Include the following details if this study involves the collection of plant, animal, or other materials from a natural setting:<br>• Field permit number<br>• Name of the institution or relevant body that granted permission	
**Data Availability**<br><br>Authors are required to make all data underlying the findings described fully available, without restriction, and from the time of publication. PLOS allows rare exceptions to address legal and ethical concerns. See the PLOS Data Policy and FAQ for detailed information.	No - some restrictions will apply

Powered by Editorial Manager® and ProduXion Manager® from Aries Systems Corporation

A Data Availability Statement describing where the data can be found is required at submission. Your answers to this question constitute the Data Availability Statement and **will be published in the article**, if accepted.<br><br>**Important:** Stating 'data available on request from the author' is not sufficient. If your data are only available upon request, select 'No' for the first question and explain your exceptional situation in the text box.<br><br>Do the authors confirm that all data underlying the findings described in their manuscript are fully available without restriction?	
**Describe where the data may be found in full sentences. If you are copying our sample text, replace any instances of XXX with the appropriate details.**<br><br>• If the data are **held or will be held in a public repository**, include URLs, accession numbers or DOIs. If this information will only be available after acceptance, indicate this by ticking the box below. For example: *All XXX files are available from the XXX database (accession number(s) XXX, XXX.).*<br>• If the data are all contained **within the manuscript and/or Supporting Information files**, enter the following: *All relevant data are within the manuscript and its Supporting Information files.*<br>• If neither of these applies but you are able to provide **details of access elsewhere**, with or without limitations, please do so. For example:<br><br>*Data cannot be shared publicly because of [XXX]. Data are available from the XXX Institutional Data Access / Ethics Committee (contact via XXX) for researchers who meet the criteria for access to confidential data.*<br><br>*The data underlying the results presented in the study are available from (include the name of the third party*	All relevant data are within the manuscript and its Supporting Information files

and contact information or URL).<br>• This text is appropriate if the data are owned by a third party and authors do not have permission to share the data.<br><br>* typeset	
Additional data availability information:	Tick here if your circumstances are not covered by the questions above and you need the journal’s help to make your data available.

Powered by Editorial Manager® and ProduXion Manager® from Aries Systems Corporation

Manuscript Click here to access/download;Manuscript;sicklecheck final manuscript 02022024.docx

Title: Evaluation of a point-of-care rapid diagnostic test kit (SICKLECHECK^{TM}) for screening of sickle cell disorders

List of Authors

Prasanta Purohit[1], Chinmayee Parida[1]; Tapan Kumar Martha[1]; Snehal Bholo[1]; Aparupa Naik[1]; Samira Kumar Behera[1,2*]

[1]Multi-Disciplinary Research Unit, Maharaja Krishna Chandra Gajapati (M.K.C.G) Medical College, Berhampur, Odisha, India

[2]Department of Pathology, Maharaja Krishna Chandra Gajapati (M.K.C.G) Medical College, Berhampur,Odisha, India

***Corresponding Author**

Dr Samira Kumar Behera
Associate Professor,
Department of Pathology,
M.K.C.G. Medical College, Berhampur
Mail ID: samirbehera41@gmail.com
Mob No. +91-94370-67376

Title: Evaluation of a point-of-care rapid diagnostic test kit (SICKLECHECK^{TM}) for screening of sickle cell disorders

Abstract:

Sickle cell disorders (SCD) are the most common genetic disorders with significant morbidity and mortality worldwide, including in India. The high prevalence of this disorder in many geographical regions calls for the use of a point-of-care rapid diagnostic test (RDT) for early screening and management of the diagnosed cases to reduce the allied clinical severity. In view of this, the present study was undertaken for the validation of a point-of-care RDT kit (SICKLECHECK^{TM}) for the screening of SCD. This validation and diagnostic accuracy study was conducted among the cases advised for screening of SCD. For validation, all the recruited cases were investigated for both the SICKLECHECK^{TM} RDT kit and HPLC (Variant-II) considering HPLC as a gold standard. A total of 400 cases were screened for both tests. For the presence and absence of sickle cell hemoglobin in the samples, SICKLECHECK^{TM} RDT kit results showed a sensitivity and specificity of 99.39% and 98.73% respectively with references to HPLC findings. For the detection of the 'AS' pattern, the SICKLECHECK^{TM} RDT kit has shown a sensitivity and specificity of 99.07% and 98.81% respectively. For the detection of the 'SS' pattern, the SICKLECHECK^{TM} RDT kit has shown a sensitivity and specificity of 97.92% and 100.0% respectively. Cases with β thalassemia trait, hemoglobin E trait, hemoglobin Lepore trait and trait for hereditary-persistence-of-fetal-hemoglobin (high HbF %) diagnosed in HPLC were resulted with 'AA' pattern in SICKLECHECK^{TM} RDT kit. The high sensitivity and specificity of the SICKLECHECK^{TM} RDT kit insist on its use as a point-of-care screening tool for SCD especially where there is a lack of laboratory facilities as well as in hospital-based set-up requiring immediate diagnosis and management of SCD. However, for further confirmation, the samples should be analyzed with other gold standard techniques like HPLC.

Keywords: Sickle Cell Disorder; SICKLECHECK^{TM}; Rapid Diagnostic Test; Early Diagnosis

Introduction

Sickle cell disorder (SCD) is one of the most common monogenic inherited diseases in the world and adds more than 0.25 millionnew cases each year.[1] In 2009, SCD was recognized as a public health problem by the WHO. With significant morbidity and mortality, SCD is also a major health burden in India contributing about 15% of SCD newborns of the world as well as ranked as the second worst affected country for SCD globally.[2,3] In India, the prevalence of SCD is mainly specific to certain populations residing in various states including Odisha, Chhattisgarh, Jharkhand, Madhya Pradesh, Maharashtra and Gujarat. Further, SCD is predominantly prevalent in socio-economically disadvantaged ethnic groups like the Scheduled Tribes, the Scheduled Castes and Other Backward Class communities in India.[4,5] Overall, the prevalence of the sickle cell gene in India varies from 1-40% in various communities.

Sickle cell disorders are defined as the presence of sickle hemoglobin (HbS), which is a structural variant of normal adult hemoglobin (Hb A) caused by a point mutation in the 20th nucleotide of β globin gene replacing glutamic acid by valine at 6th aminoacid (*HBB*; c.20T>A, p.Glu6Val; OMIM: 141900 (*HBB*-β^{S})].[6] This Hb S polymerizes under low oxygen conditions leading to the formation of a crescent or sickle shaped red blood cells. The inheritance of Hb S occurs either in the heterozygous condition (sickle cell trait/sickle cell carrier/AS) or in homozygous condition (homozygous sickle cell anemia/SCA/SS) or in double heterozygous conditions with other hemoglobin variants. The ‘AS’ individuals are usually asymptomatic whereas both ‘SS’ individuals and double heterozygous state with Hb S are characterized by asymptomatic to severe clinical manifestations. The most clinical presentations are episodes of painful events, anemia requiring blood transfusion, acute chest syndrome, jaundice, stroke etc.[7,8]

At present many methods are there for the diagnosis of SCD including the sickling slide test, solubility test, Hb electrophoresis, Isoelectric focusing, HPLC and genetic testing.[9-11] Most of these methods can only be used in laboratories requiring a supply of electricity, trained manpower as well as the maintenance of equipment which is very difficult in resource-poor setting especially when carried out in tribal-dominated areas. For the early diagnosis as well as management of SCD, now the world is focused on point-of-care test.[12] A number of point-of-care rapid diagnostics test (RDT) kits are available for the diagnosis of SCD including HemoTypeSC™, SickleScan®, SickleDex, solubility test etc. with significant sensitivity and specificity. The use of RDT kits for the screening of SCD will be helpful for the early identification of SCD patients and the subsequent provision of comprehensive

management to reduce the morbidity and mortality rate related to SCD. Further, the use of RDT kits does not require any sophisticated laboratory set-up. In this regard, the present study was undertaken for the validation as well as the usefulness of another point-of-care RDT kit (SICKLECHECK^{TM}) for the screening of SCD.

Materials and Methods

This was a validation, diagnostic accuracy study of the SICKLECHECK^{TM} RDT kit conducted among the patients attending Maharaja Krishna Chandra Gajapati (M.K.C.G) Medical College for various health problems and advised for screening of SCD. This is a tertiary health care center with many specialists and caters to around 13 million people residing in the southern districts of the state of Odisha (19.3077° N, 84.8110° E). As a routine screening procedure for hemoglobin disorders including SCD, patients attending various departments are advised for HPLC for the screening of hemoglobin disorders to the Department of Pathology, M.K.C.G. Medical College. In association with the Department of Pathology, this study was carried out in the Multi-Disciplinary Research Unit of M.K.C.G. Medical College. This research unit has been established by the Department of Health Research (DHR), Ministry of Health and Family Welfare, Government of India to strengthen the research activities, especially for non-communicable diseases in various health institutions in India.

This study was carried out in individuals attending this hospital from June 2023 to August 2023. In an earlier hospital-based study carried out in this Medical College, the sickle cell disorder was reported in around 50% of cases.[13] Considering the hypothesized % frequency of outcome factor in the population of 50%, confidence level of 95% and absolute error of 5%, the sample size calculated as 384 (fixed to 400).

After obtaining written informed consent from the patients (from the parents/guardian in case of a minor), 1-2 mL of venous blood samples were collected in an EDTA vial for laboratory investigations. All the collected blood samples were analyzed for both HPLC (Variant-II, Bio-Rad Laboratories, CA, USA) using β thalassemia short program and a rapid diagnostic test kit (SICKLECHECK^{TM}, Zephyr Biomedicals- A division of Tulip Diagnostics (P) Ltd., Goa, India) for the screening of SCD simultaneously. This study was approved by the Institutional Ethical Committee of M.K.C.G Medical College, Berhampur, Odisha (Ref No 1298/Chairman-IEC, M.K.C.G. Medical College, Brahmapur-4, Date 05. 04.2023)

SICKLECHECK^{TM} RDT kit is a point-of-care, rapid, qualitative, immuno-chromatographic assay for the simultaneous detection of Hb S and Hb A in human blood

samples. It is based on the principle of agglutination of antibodies/antisera with respective antigens in a competitive immuno-chromatography format along with the use of nanogold particles as agglutination revealing agents. The designed test kit pad is impregnated with two components that is, monoclonal antibody for Hb S conjugated to colloidal gold and monoclonal antibody for Hb A conjugated to colloidal gold. As the test sample flows through the membrane assembly of the kit, the highly specific monoclonal antibody for Hb S and Hb A conjugated with colloidal gold make complexes with the respective Hb S and Hb A antigens present in the test sample and travels on the membrane due to capillary action. The complex travels on the membrane to the test region (S and A mark in the kit) where it is not captured by Hb S and Hb A coated on the membrane, thus forming no bands. The absence of colour bands at the test regions (S and A) indicates the presence of respective Hb S and Hb A antigens in the test samples and vice versa. The unbound colloidal gold conjugates move further on the membrane and are immobilized by the agglutinating sera for goat anti-mouse IgG coated on the membrane at the control (C mark in the kit). This control band acts as a procedural control and serves to validate the test results. The detailed interpretation of results generated from theSICKLECHECK^{TM} RDT kit is shown in Figure 1.

This SICKLECHECK^{TM} RDT kit contains membrane test assembly, plastic specimen transfer devices with a 10 µL mark, assay buffer vials and sterile lancets. The test can be performed either in venous blood or by finger prick. In both case, 10 µL of blood samples was mixed with supplied assay buffers by gently shaking the vial sideways. Two drops of the specimen were applied into the specimen port (B) of the membrane device and after 10-15 minutes the result was interpreted by observing the clear bands in the C, S and A regions respectively. Step-wise pictorial procedure for the SICKLECHECK^{TM} RDT kit application is illustrated in Figure 2.

Data analysis

The characteristics of the study participants were described using descriptive statistics. The generated results of both test were analyzed by respective instruction manuals and categorized the cases accordingly. A representative set of SICKLECHECK^{TM} RDT kits analyzed during this study is provided as Figure 3. The diagnostic accuracy of the SICKLECHECK^{TM} RDT kit was compared with HPLC results as the gold standard by analyzing sensitivity, specificity, positive predictive value (PPV) and negative predictive value (NPV) using 2023 MedCalc Software Ltd.(http://www.medcalc.org/calc/diagnostic_test.php).

First, the diagnostic accuracy of the SICKLECHECK^{TM} RDT kit was performed by analyzing the presence and absence of Hb S in the samples as follows

1. For HPLC
   - Presence of Hb S: Cases with 'AS', 'SS', sickle cell and β thalassemia (HbS- β thalassemia), and SS with a history of blood transfusion.
   - Absence of Hb S: Cases with 'AA', 'β thalassemia trait', 'β thalassemia major', 'hemoglobin E trait', 'hemoglobin Lepore trait', and 'trait for hereditary persistence of fetal hemoglobin'.
2. For SICKLECHECK^{TM} RDT kit
   - Presence of Hb S: Cases with 'AS' and 'SS' pattern
   - Absence of Hb S: Cases with 'AA' and 'Others' (other hemoglobin variants).

Secondly, as the SICKLECHECK^{TM} RDT kit is based on the screening for the SCD only, cases with 'AA', 'AS' and 'SS' patterns detected in HPLC were only considered for the diagnostic accuracy of the SICKLECHECK^{TM} RDT kit.

Results

During the study period, a total of 400 unknown samples were included in this study. The median age of the study participants was 19 years with a range from 7 months to 70 years. There were 204 females and 196 males. For the diagnosis of SCD in all the recruited cases both HPLC (Variant-II) and SICKLECHECK^{TM} RDT were carried out simultaneously. In HPLC, along with 'AA', 'AS' and 'SS' pattern, other variants were also detected including double heterozygous state of Hb S and β thalasemia (4 cases), hemoglobin E disorder (1 case), Hemoglobin Lepore (1 case), hereditary persistence of fetal Hemoglobin (7 cases), β thalasemia trait (20 cases), β thalasemia major (3 cases) etc. In SICKLECHECK^{TM} RDT, a total of 228, 110, 56 and 6 cases were detected as 'AA', 'AS', 'SS' and 'others' patterns respectively. The detailed distribution of cases on the basis of HPLC and SICKLECHECK^{TM} RDT results is depicted in Table 1.

On comparison of both the investigations, various hemoglobin disorders like normal (AA), β thalassemia trait, hemoglobin E trait, hemoglobin Lepore trait and trait for hereditary persistence of fetal hemoglobin (high HbF %) diagnosed in HPLC were resulted with 'AA' pattern in SICKLECHECK^{TM} RDT kit. Similarly, the'AS' pattern and trait for hereditary persistence of foetal hemoglobin (high HbF %) diagnosed in HPLC resulted in the 'AS' pattern in the SICKLECHECK^{TM} RDT kit. Cases with HbS-β thalassemia, 'SS' with a history of blood transfusion and 'SS' in HPLC resulted in 'SS' pattern in the

SICKLECHECK^{TM} RDT kit. All the β thalassemia major cases in HPLC were detected as 'Others' pattern in the SICKLECHECK^{TM} RDT kit. The detailed comparative analysis of both HPLC and SICKLECHECK^{TM} RDT kit is depicted in Table 2.

As per HPLC finding, Hb S was present in 164 cases and absent in rest 236 cases. Out of 164 cases with Hb S in HPLC, 163 cases had Hb S and a lone case had no Hb S in the SICKLECHECK^{TM} RDT kit. Similarly, out of 236 cases with no Hb S in HPLC, 233 cases had no Hb S and 3 cases had Hb S in the SICKLECHECK^{TM} RDT kit. With references to HPLC findings, SICKLECHECK^{TM} RDT kit results showed a sensitivity of 99.39% and a specificity of 98.73% (Figure 4).

As the SICKLECHECK^{TM} RDT kit is designed for the diagnosis of SCD only, we have calculated the sensitivity and specificity from 359 cases including 204 cases of the 'AA' pattern, 107 cases of the 'AS' pattern and 48 cases of the 'SS' pattern resulted in HPLC. The detailed distribution of 359 cases is shown in Table 3. The sensitivity and specificity of the SICKLECHECK^{TM} RDT kit were analyzed separately for the 'AA', 'AS' and 'SS' pattern. For the detection of the 'AS' pattern, the SICKLECHECK^{TM} RDT kit has shown a sensitivity of 99.07% and a specificity of 98.81%. For the detection of the 'SS' pattern, the SICKLECHECK^{TM} RDT kit has shown a sensitivity of 97.92% and a specificity of 100.0%. For the detection of the 'AA' pattern, the SICKLECHECK^{TM} RDT kit has shown a sensitivity of 98.04% and a specificity of 99.35%. The sensitivity, specificity, PPV and NPV of the SICKLECHECK^{TM} RDT kit for the detection of sickle cell disease are depicted in Table 4.

In the comparison of both the HPLC and SICKLECHECK^{TM} RDT kit in 359 cases, 6 cases showed conflicting results with respect to each test. As the SICKLECHECK^{TM} RDT kit has certain limitations for lower hemoglobinlevel, high fetal hemoglobin level, percentage of both Hb S and Hb A in the samples etc., the detailed hemoglobin level along with HPLC finding of 6 contradictory cases is shown in Table 5. None of these cases had significant findings that can be correlated with the limitations of the SICKLECHECK^{TM} RDT kit.

Discussion

During this validation study, the result of SICKLECHECK^{TM} RDT kit was compared with the HPLC as a gold standard technique for the screening of SCD. In comparison, the SICKLECHECK^{TM} RDT kit was found to have high sensitivity and specificity for the screening of SCD. It showed high sensitivity and specificity of more than 98% in both when compared to either the presence or absence of Hb S in the samples; or the genotypes (AA, AS, SS) of the individuals respectively. These observations insist on the use of the SICKLECHECK^{TM} RDT kit as a screening tool for SCD.

SICKLECHECK^{TM} RDT kit can be used in remote areas without requiring sophisticated equipment, electricity, laboratory etc. Only a trained person can perform the test as well as interpret the results easily. Further, the results can be read within 15 minutes of the blood collection affirming its use in many hospital set-ups necessitating immediate diagnosis of the SCD for proper management and treatment. In the state of Odisha with high prevalence of SCD as well as inhabitants of many tribal communities especially residing in remote areas with minimal or no health facilities, the SICKLECHECK^{TM} RDT kit will be useful as a point-of-care test for SCD. The use of point-of-care RDT for the screening of SCD will lead to early identification of disease and subsequent provision of comprehensive care. The comprehensive care management of SCD patients especially during childhood has been associated with a significant reduction in morbidity and mortality as well as most of the affected children attended their adulthood.[14]

During the study, a number of other Hb variants were also reported and some of them were also co-inherited with Hb S. Interestingly, all the double heterozygous states of HbS and β thalassemia diagnosed by HPLC were reported as ‘SS’ patterns in SICKLECHECK^{TM} RDT kit. Again, all four ‘SS' cases with a history of blood transfusion (HbA0 level was 10-16% in HPLC) were also reported as ‘SS’ pattern in the SICKLECHECK^{TM} RDT kit. Both of these observations also affirm its use in other variants of SCD including double heterozygous state of HbS and β thalassemia which is the second most prevalent severe phenotype of SCD after ‘SS’ genotype worldwide including India.[15] However, its pattern in another double heterozygous state with HbS needs to be investigated further concerning the high prevalence of Hb variant types with respect to different geographical areas.

This SICKLECHECK^{TM} RDT kit has also been designed for the diagnosis of other Hb variants. Except few, the majority of Hb variants including β thalassemia trait, hemoglobin E trait, hemoglobin Lepore trait and trait for hereditary persistence of fetal hemoglobin (high HbF %) diagnosed by HPLC in this study were resulted with ‘AA’ pattern in SICKLECHECK^{TM} RDT kit. All three β thalassemia major cases in HPLC were detected as ‘Others’ patterns in the SICKLECHECK^{TM} RDT kit. Thus, the diagnosis of other Hb variants (other than Hb S) by SICKLECHECK^{TM} RDT kit needs to be revised requiring further upgradation of membrane biology. In such a finding, the samples should be analyzed by HPLC for further confirmation.

The use of competitive immuno-chromatography assay in the SICKLECHECK^{TM} RDT kit enhances the specificity of the test results by avoiding the possible interferences from other proteins in the samples as well as can allow the detection of a wide range of

concentrations of target molecules (Hb A and Hb S). It may also exhibit lower background signals in the absence of the target Hb, which can contribute to improve the signal-to-noise ratios resulting better sensitivity in certain situations. Further this competitive immunoassay may be a better option from the sandwich type immunoassay which are usually susceptible to the prozone effect (hook effect), in which a high concentrations of a particular Hb may leading to a saturation of the binding sites on the labelled conjugate and capture reagent on the test line, as a result, the excess Hb competes less effectively with the labelled conjugate, causing a decrease in the signal at the test line yielding a false negative results.[16-18]

There are certain limitations of the study; (1) it does not quantify the amount of Hb S and Hb A in the samples; (2) The presence of heterophilic substances in the samples and lipemic or icteric samples may give incorrect results though no such type of samples was analyzed in this study. (3) The presence of a control line only means that the migration of the specimen is occurred. It does not serve as confirmation of the addition of more test specimens.

In conclusion, the SICKLECHECK^{TM} RDT kit can be used as a point-of-care test for the screening of SCD only. The readability of results in the SICKLECHECK^{TM} RDT kit is easy and clear. A clear and dark band in the test membrane should be considered for diagnosis; while the appearance of any faint or light bands should go for further confirmation with other gold standard techniques like HPLC.

Funding details: This study was funded by Department of Health Research, Ministry of Health and Family Welfare, Government of India (Letter No. V.25011/570(i)/2010-HR Dt.07.01.2014) for the establishment of a Multi-Disciplinary Research Unit (MRU) at Maharaja Krishna Chandra Gajapati (M.K.C.G.) Medical College, Berhampur, Odisha, India.

Acknowledgement: The authors acknowledge laboratory technician, Mrs Radha Rani Dora and Mr. Himansu Sekhar Sahu for their support in collection and processing of blood samples during the investigations. The authors also acknowledge to Mr. Satyanarayana Mohanty, Data Entry Operator, Multi-Disciplinary Research Unit (MRU) for data processing. For use of the SICKLECHECK^{TM} Kit information or photographs, necessary permission has been obtained from the Zephyr Biomedicals- A division of Tulip Diagnostics (P) Ltd., Goa, India.

Conflict of Interest: None

Author's contribution: PP and SKB designed the research; PP, CP, TKM and SB performed all the laboratory analysis; PP and SKB interpreted the results; PP performed all the statistical analysis; PP, AN and SKB wrote the manuscript; All the authors gave final approval of the manuscript for publication.

References

1. Modell B, Darlison M. Global Epidemiology of Haemoglobin Disorders and Derived Service Indicators. Bull World Health Organ. 2008;86(6):480-487.
2. Piel FB, Hay SI, Gupta S, Weatherall DJ, Williams TN. Global burden of sickle cell anaemia in children under five, 2010-2050:modelling based on demographics, excess mortality, and interventions. PLoS Med 2013;10:e1001484.
3. Hockham C, Bhatt S, Colah R, et al. The spatial epidemiology of sickle cell anemia in India. Sci Rep. 2018;8:17685.
4. Shrikhande AV, Arjunan A, Agarwal A, Dani A, Tijare J, Gettig E, Krishnamurti L. Prevalence of the β(S) gene among scheduled castes, scheduled tribes and other backward class groups in Central India. Hemoglobin. 2014;38(4):230-5.
5. Colah RB, Mukherjee MB, Martin S, Ghosh K. Sickle cell disease in tribal populations in India. Indian J Med Res. 2015;141:509–15.
6. Esoh K, Wonkam A. Evolutionary history of sickle-cell mutation: implications for global genetic medicine. Hum Mol Genet. 2021;30(R1):R119-R128.
7. Eaton WA, Hofrichter J. Sickle cell haemoglobin polymerization. Adv Protein Chem. 1990;40:63–279
8. Makani J, Ofori-Acquah SF, Nnodu O, Wonkam A, Ohene-Frempong K. Sickle cell disease: new opportunities and challenges in Africa. Scientific World Journal 2013; 2013:193252.
9. Wild B, Stephens A. The use of automated HPLC to detect and quantitate haemoglobins. Clinical & Laboratory Haematology, 1997. 19(3): 171-176
10. Jenkins MA, Ratnaike S. Capillary isoelectric focusing of haemoglobin variants in the clinical laboratory. Clin Chim Acta 1999; 289: 121-32
11. Clarke GM, Higgins TN. Laboratory investigation of hemoglobinopathies and thalassemias: review and update. Clin Chem. 2000;46(8 Pt 2):1284-90.
12. Ilyas S, Simonson AE, Asghar W. Emerging point-of-care technologies for sickle cell disease diagnostics. Clin Chim Acta. 2020 Feb;501:85-91.
13. Sahu P, Purohit P, Mantri S, Tudu R, Nayak J, Agrawalla SK, et al. Incidence of hemoglobin disorders in southern Odisha, India: A hospital based study. Porto Biomed J 2021; 6(1):e126

14. Makani J, Soka D, Rwezaula S, et al. Health policy for sickle cell disease in Africa: experience from Tanzania on interventions to reduce under-five mortality. Trop Med Int Health. 2015;20(2):184-187.
15. Dehury S, Purohit P, Patel S, et al. Low and fixed dose of hydroxyurea is effective and safe in patients with HbSβ^{+} thalassemia with IVS1-5(G→C) mutation. Pediatric Blood Cancer. 2015;62(6).1017-1023.
16. Fernando SA, Wilson GS. Studies of the “hook” effect in the one-step sandwich immunoassay. J Immunol Methods. 1992; 151:47–66
17. Bond M, Hunt B, Flynn B, Huhtinen P, Ware R, Richards-Kortum R. Towards a point-of-care strip test to diagnose sickle cell anemia. PLoS ONE 2017;12(5): e0177732
18. Steele C, Sinski A, Asibey J, Hardy-Dessources M, Elana G, Brennan C et al. Point-of-care screening for sickle cell disease in low-resource settings: A multi-center evaluation of HemoTypeSC, a novel rapid test. Am J Hematol. 2019;94:39–45

List of Tables

Table 1: Distribution of cases on the basis of HPLC (Variant-II) and SICKLECHECK™ RDT kit results (n=400).

Table 2: Comparison of results from HPLC (Variant II) and SICKLECHECK™ RDT kit (n=400)

Table 3: Distribution of cases with respect to HPLC (Variant-II) and SICKLECHECK™ RDT kit results (n=359).

Table 4: SICKLECHECK™ RDT kit sensitivity, specificity, positive predictive value and negative predictive value in comparison to HPLC findings for the detection of sickle cell disorders.

Table 5: Cases with conflicting results (n=6).

List of the figures

Figure 1: Interpretation of results of SICKLECHECK^{TM} Rapid Diagnostic Kit for the diagnosis of sickle cell disorders.

Figure 2: Step-wise pictorial procedure for SICKLECHECK^{TM} Rapid Diagnostic Kit for the diagnosis of sickle cell disorders.

Figure 3: A set of SICKLECHECK^{TM} RDT kits analyzed during the study.

Figure 4: Sensitivity and specificity of SICKLECHECK^{TM} RDT kit for the presence of sickle hemoglobin.

Table 1: Distribution of cases on the basis of HPLC (Variant-II) and SICKLECHECK™ RDT kit results (n=400).

Hemoglobin types	HPLC (Variant-II) (Number)	SICKLECHECK™ RDT (Number)
AA	204	228
AS	107	110
SS	48	56
SS with history of blood transfusion	4	0
β thalassemia trait	20	0
HbS-β thalassemia	5	0
HbE trait	1	0
Hb Lepore trait	1	0
β thalassemia major	3	0
Hereditary persistence of fetal Hemoglobin	7	0
Others	0	6

Table 2: Comparison of results from HPLC (Variant II) and SICKLECHECK^{TM} RDT kit (n=400)

**SICKLECHECK^{TM} RDT kit**	**HPLC (Variant-II)**
AA (n=228)	AA (n=200)
	AS (n=1)
	β thalassemia trait (n=20)
	Hemoglobin E trait (n=1)
	Hemoglobin Lepore trait (n=1)
	Trait for hereditary persistence of fetal hemoglobin (n=5)
AS (n=110)	AA (n=2)
	AS (n=106)
	Trait for hereditary persistence of fetal hemoglobin (n=1)
	SS (n=1)
SS (n=56)	SS (n=47)
	S-β thalassemias (n=5)
	SS with history of blood transfusion (n=4)
Others (n=6)	AA (n=2)
	β thalassemia major (n=3)
	Trait for hereditary persistence of fetal hemoglobin (n=1)

Table 3: Distribution of cases with respect to HPLC (Variant-II) and SICKLECHECK^{TM} RDT kit results (n=359).

**Sickle Cell Disorders**		**SICKLECHECK^{TM} RDT kit**				**Total**
		**‘AA’ pattern**	**‘AS’ pattern**	**‘SS’ pattern**	**‘Others’ pattern**	
**HPLC Results**	**AA**	**200**	**2**	**0**	**2**	**204**
	**AS**	**1**	**106**	**0**	**0**	**107**
	**SS**	**0**	**1**	**47**	**0**	**48**
**Total**		**201**	**109**	**47**	**2**	**359**

Table 4: SICKLECHECK^{TM} RDT kit sensitivity, specificity, positive predictive value and negative predictive value in comparison to HPLC findings for the detection of sickle cell disorders.

	**SICKLECHECK^{TM} RDT kit**					
	**'AA' pattern**		**'AS' pattern**		**'SS' pattern**	
	Value	95% CI	Value	95% CI	Value	95% CI
**Sensitivity**	98.04	95.06-99.46	99.07	94.90-99.98	97.92	88.93-99.95
**Specificity**	99.35	96.46-99.98	98.81	96.56-99.75	100.00	98.82-100.00
**Positive predictive value**	99.50	97.26-99.99	97.25	92.17-99.43	100.00	92.45-100.0
**Negative predictive value**	97.47	93.65-99.31	99.60	97.79-99.99	99.68	98.23-99.99

Table 5: Cases with conflicting results (n=6).

Case No.	Results of SICKLECHECK™ RDT kit	Results of HPLC	Hemoglobin variants from HPLC (%)				Total hemoglobin level (mg/dL)
			HbA0	HbA2	HbF	HbS	
1	AS	AA	84.5	2.6	0.6	0	11.3
2	AS	AA	80.1	2.7	0.4	0	7.7
3	AS	SS	6.5	2.7	14.3	73.3	7.3
4	Other	AA	88.1	3.2	0.9	0	11.9
5	AA	AS	67.4	3.8	4.1	17.0	11.7
6	Other	AA	84.2	2.7	1.8	0	10.9

Figure 1: Interpretation of results of SICKLECHECKTM Rapid Diagnostic Kit for the diagnosis of sickle cell disorders.

Click here to access/download;Figure;Figure 1.jpeg

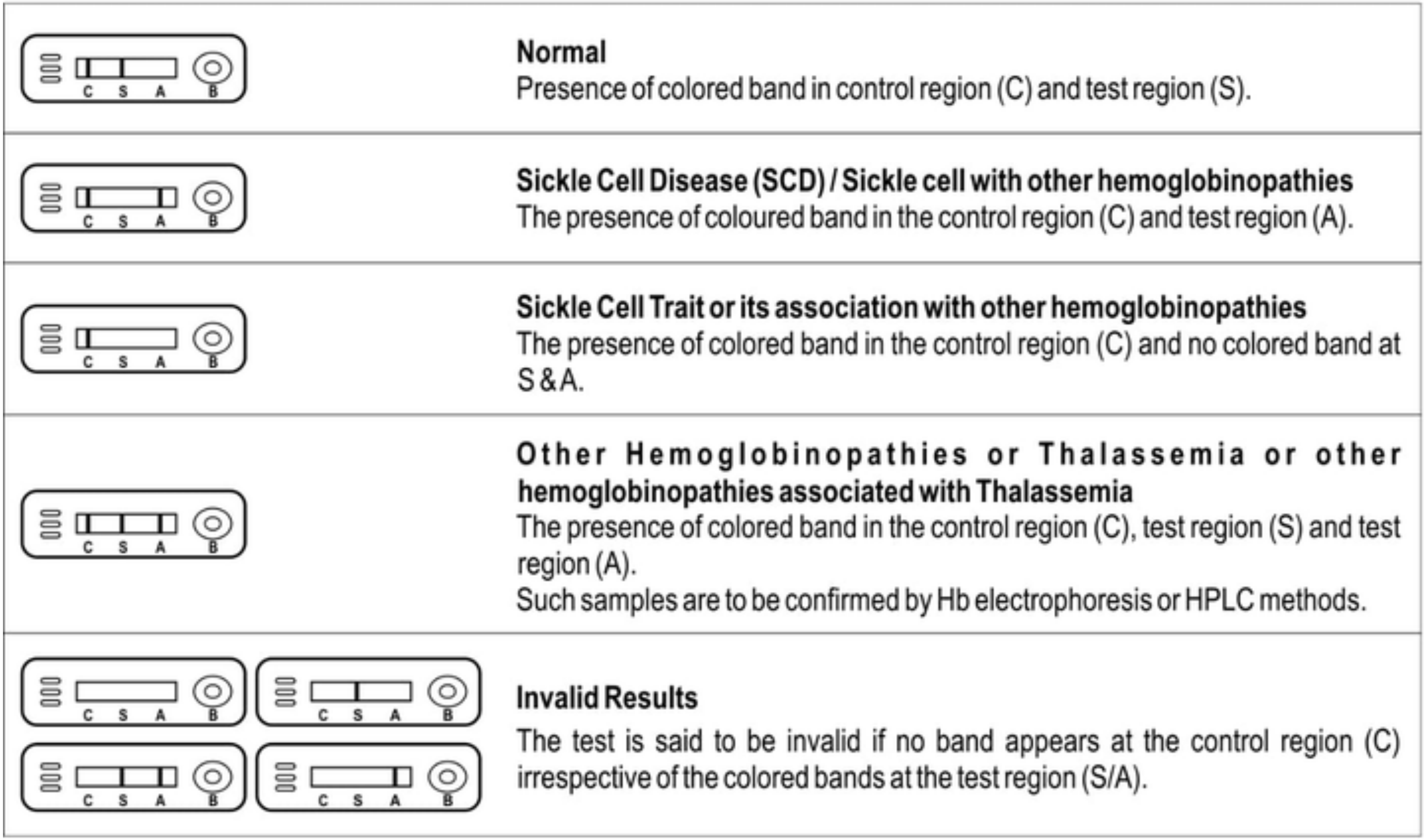

Figure 2: Step-wise pictorial procedure for SICKLECHECKTM Rapid Diagnostic Kit for the diagnosis of sickle cell disorders

Click here to access/download;Figure;Figure 2.jpeg

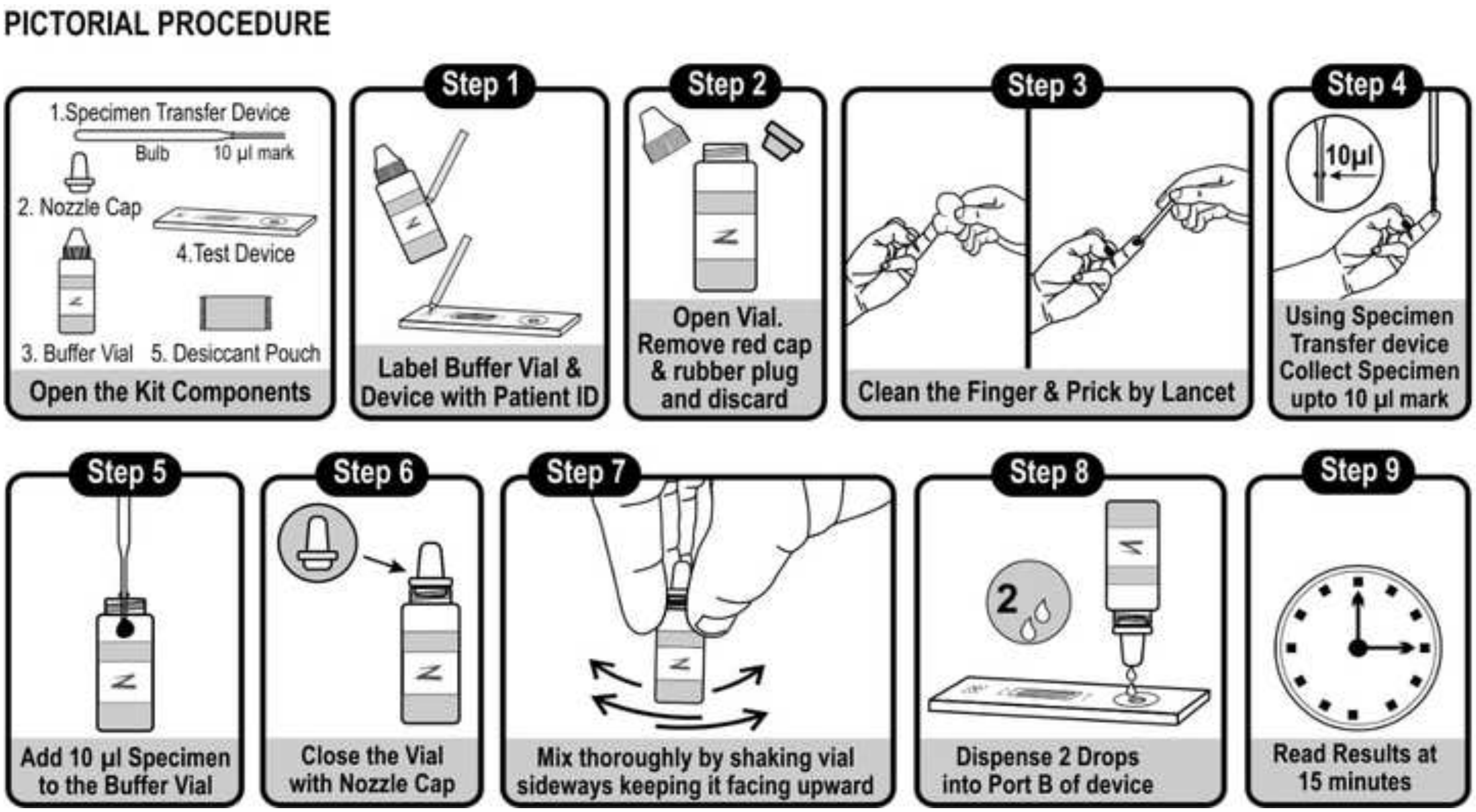

Figure 3: A set of SICKLECHECKTM RDT kits analyzed during the study.

Click here to access/download;Figure;Figure 3.tif

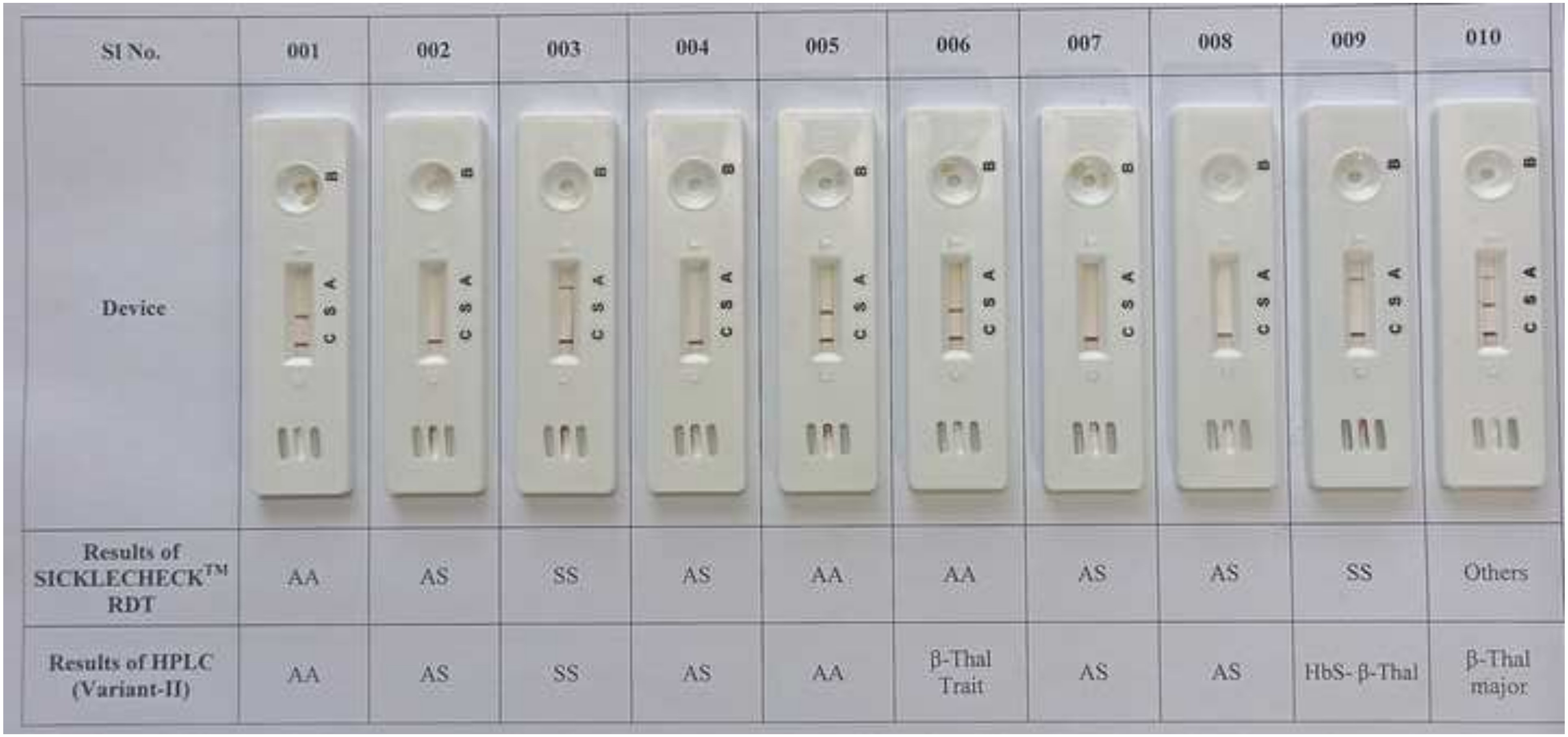

Sl No.	001	002	003	004	005	006	007	008	009	010
Device										
Results of SICKLECHECK™ RDT	AA	AS	SS	AS	AA	AA	AS	AS	SS	Others
Results of HPLC (Variant-II)	AA	AS	SS	AS	AA	β-Thal Trait	AS	AS	HbS- β-Thal	β-Thal major

Figure 4: Sensitivity and specificity of SICKLECHECKTM RDT kit for the presence of sickle hemoglobin.

Click here to access/download;Figure;Figure 4.tif

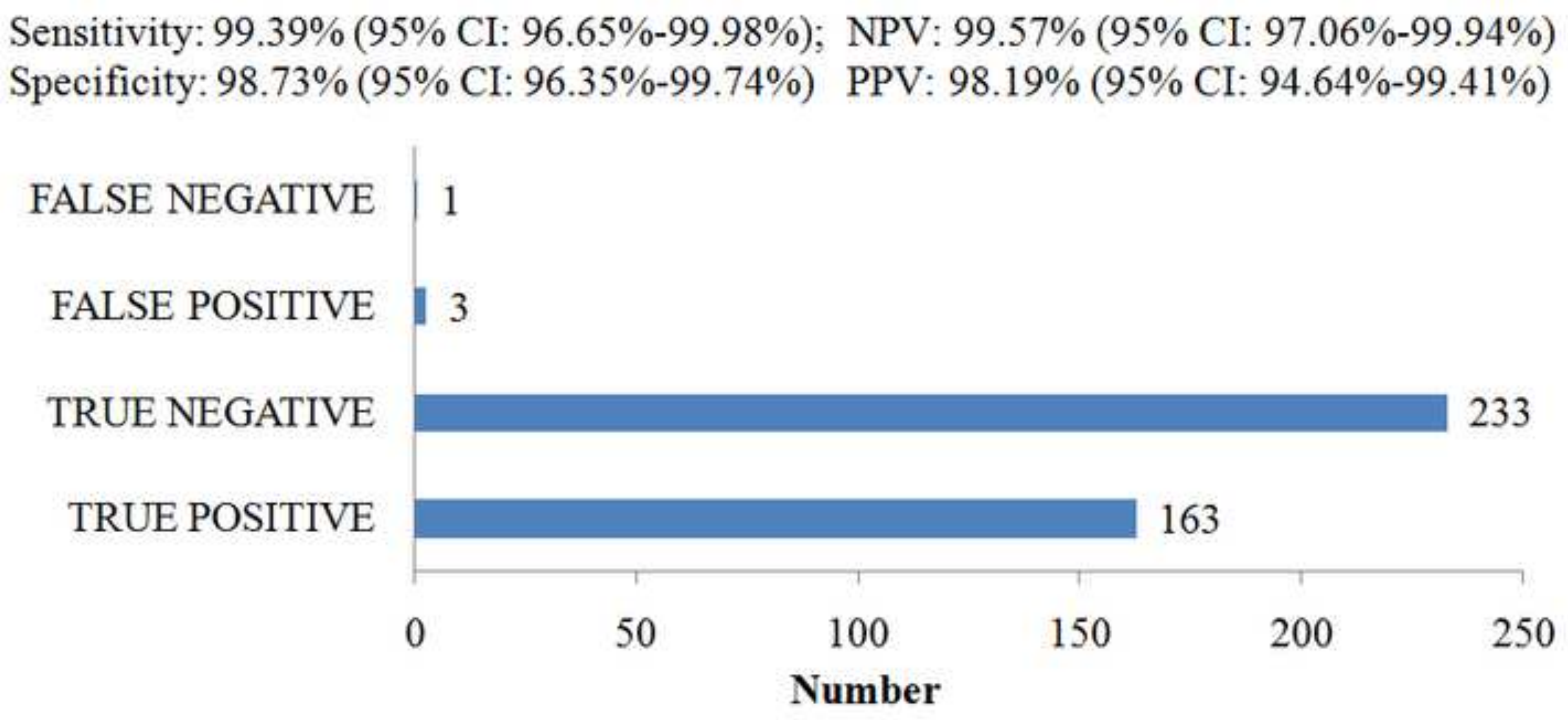

Manuscript ID : PONE-D-24-04629
Title: Evaluation of a point-of-care rapid diagnostic test kit (SICKLECHECKTM) for screening of sickle cell disorders

Authors Responses

1. In consent please state in Ethics Method section and manuscript if it is written or verbal. If consent was verbal, please explain a) why written consent was not obtained, b) how you documented participant consent, and c) whether the ethics committees/IRB approved this consent procedure.

   Response: Written informed consent from all the participants was obtained. In case of minors, the written informed consent was obtained from their parents/ guardians. This has also been included in the manuscript.

2. Please complete all items on the Human Participants Research Checklist that are relevant for your submission, by following this link: http://journals.plos.org/plosone/s/file?id=dc11/PLOSOne_Human_Subjects_Research_Checklist.docx (Contact us at plosone@plos.org if you cannot access the document.) There may be overlap between the checklist items and other queries listed below; please address any duplicated queries both in your response email and on the checklist itself. Upload the completed Human Participants Research Checklist as file type "Other" when you re-submit your manuscript. This document is for internal journal use only and will not be published if your article is accepted. The requested information will help us to assess whether your submission complies with PLOS ONE's policies and adheres to applicable reporting standards. Note that your manuscript may be rejected if you provide incomplete or inadequate responses to the checklist questions and that changing the 'Section/Category' of your article does not affect this requirement.

   Response: Submitted to the journal.
