# Supplementary material for: Evaluation of a point-of-care rapid diagnostic test kit (SICKLECHECK) for screening of sickle cell diseases
Source: PLoS One. 2024 Aug 16;19(8):e0309045. doi: 10.1371/journal.pone.0309045 (PMC11329129; doi:10.1371/journal.pone.0309045)
Supplement: Attachment — Submitted filename: Compliance to Reviewers.docx [file pone.0309045.s002.docx]

Manuscript ID : PONE-D-24-04629
Title: Evaluation of a point-of-care rapid diagnostic test kit (SICKLECHECKTM) for screening of sickle cell disorders

Authors Responses

1. In consent please state in Ethics Method section and manuscript if it is written or verbal. If consent was verbal, please explain a) why written consent was not obtained, b) how you documented participant consent, and c) whether the ethics committees/IRB approved this consent procedure.

**Response:** Written informed consent from all the participants was obtained. In case of minors, the written informed consent was obtained from their parents/ guardians. This has also been included in the manuscript.

2. Please complete all items on the Human Participants Research Checklist that are relevant for your submission, by following this link:  <http://journals.plos.org/plosone/s/file?id=dc11/PLOSOne_Human_Subjects_Research_Checklist.docx> (Contact us at [plosone@plos.org](mailto:plosone@plos.org) if you cannot access the document.) There may be overlap between the checklist items and other queries listed below; please address any duplicated queries both in your response email and on the checklist itself. Upload the completed Human Participants Research Checklist as file type “Other” when you re-submit your manuscript. This document is for internal journal use only and will not be published if your article is accepted. The requested information will help us to assess whether your submission complies with PLOS ONE’s policies and adheres to applicable reporting standards. Note that your manuscript may be rejected if you provide incomplete or inadequate responses to the checklist questions and that changing the
‘Section/Category’ of your article does not affect this requirement.

**Response:** Submitted to the journal.
