# Supplementary material for: Evaluation of a point-of-care rapid diagnostic test kit (SICKLECHECK) for screening of sickle cell diseases
Source: PLoS One. 2024 Aug 16;19(8):e0309045. doi: 10.1371/journal.pone.0309045 (PMC11329129; doi:10.1371/journal.pone.0309045)
Supplement: Attachment — Submitted filename: PLOSONE REVIEW.docx [file pone.0309045.s004.docx]

**Response to Reviewers**

PONE-D-24-04629

Evaluation of a point-of-care rapid diagnostic test kit (SICKLECHECKTM) for screening of sickle cell disorders

**Comments:** If you would like to make changes to your financial disclosure, please include your updated statement in the cover letter. If applicable, we recommend that you deposit your laboratory protocols in protocols.io to enhance the reproducibility of your results. Protocols.io assigns your protocol its own identifier (DOI) so that it can be cited independently in the future. For instructions see: <https://journals.plos.org/plosone/s/submission-guidelines#loc-laboratory-protocols>. Additionally, PLOS ONE offers an option for publishing peer-reviewed Lab Protocol articles, which describe protocols hosted on protocols.io. Read more information on sharing protocols at <https://plos.org/protocols?utm_medium=editorial-email&utm_source=authorletters&utm_campaign=protocols>.
**Response:** Thank you for suggestions, but we want to publish it along with the original research article.

Journal Requirements:

**Comments:** 1. Please ensure that your manuscript meets PLOS ONE's style requirements, including those for file naming. The PLOS ONE style templates can be found at 
https://journals.plos.org/plosone/s/file?id=wjVg/PLOSOne_formatting_sample_main_body.pdf and https://journals.plos.org/plosone/s/file?id=ba62/PLOSOne_formatting_sample_title_authors_affiliations.pdf

**Response:** Necessary modifications have been incorporated in the revised manuscript

**Comments:**

2. Thank you for stating the following financial disclosure: "This study was funded by Department of Health Research, Ministry of Health and Family Welfare, Government of India (Letter No. V.25011/570(i)/2010-HR Dt.07.01.2014) for the establishment of a Multi-Disciplinary Research Unit (MRU) at Maharaja Krishna Chandra Gajapati Medical College, Berhampur, Odisha, India."  Please state what role the funders took in the study.  If the funders had no role, please state: ""The funders had no role in study design, data collection and analysis, decision to publish, or preparation of the manuscript.""  If this statement is not correct you must amend it as needed.  Please include this amended Role of Funder statement in your cover letter; we will change the online submission form on your behalf.

**Response:** Modified as per the suggestions

**Comments:**

3. We note that you have indicated that there are restrictions to data sharing for this study. For studies involving human research participant data or other sensitive data, we encourage authors to share de-identified or anonymized data. However, when data cannot be publicly shared for ethical reasons, we allow authors to make their data sets available upon request. For information on unacceptable data access restrictions, please see http://journals.plos.org/plosone/s/data-availability#loc-unacceptable-data-access-restrictions.

Before we proceed with your manuscript, please address the following prompts:

a) If there are ethical or legal restrictions on sharing a de-identified data set, please explain them in detail (e.g., data contain potentially identifying or sensitive patient information, data are owned by a third-party organization, etc.) and who has imposed them (e.g., a Research Ethics Committee or Institutional Review Board, etc.). Please also provide contact information for a data access committee, ethics committee, or other institutional body to which data requests may be sent.

b) If there are no restrictions, please upload the minimal anonymized data set necessary to replicate your study findings to a stable, public repository and provide us with the relevant URLs, DOIs, or accession numbers. Please see http://www.bmj.com/content/340/bmj.c181.long for guidelines on how to de-identify and prepare clinical data for publication. For a list of recommended repositories, please see https://journals.plos.org/plosone/s/recommended-repositories. You also have the option of uploading the data as Supporting Information files, but we would recommend depositing data directly to a data repository if possible.

Please update your Data Availability statement in the submission form accordingly.

**Response:** Data Availability statement has been modified accordingly. Data will be available as supplementary files

**Reviewers' comments:
Comments to the Author**
1. Is the manuscript technically sound, and do the data support the conclusions?
The manuscript must describe a technically sound piece of scientific research with data that supports the conclusions. Experiments must have been conducted rigorously, with appropriate controls, replication, and sample sizes. The conclusions must be drawn appropriately based on the data presented.

Reviewer #1: Partly

Reviewer #2: Yes

**Response:** Many changes have been incorporated in the revised manuscript

**Comments:** 2. Has the statistical analysis been performed appropriately and rigorously?

Reviewer #1: No

Reviewer #2: Yes

**Response:** Necessary modifications have been incorporated in the revised manuscript

**Comments:** 3. Have the authors made all data underlying the findings in their manuscript fully available? The [PLOS Data policy](http://www.plosone.org/static/policies.action#sharing) requires authors to make all data underlying the findings described in their manuscript fully available without restriction, with rare exception (please refer to the Data Availability Statement in the manuscript PDF file). The data should be provided as part of the manuscript or its supporting information, or deposited to a public repository. For example, in addition to summary statistics, the data points behind means, medians and variance measures should be available. If there are restrictions on publicly sharing data—e.g. participant privacy or use of data from a third party—those must be specified.

Reviewer #1: Yes

Reviewer #2: No

**Response:** Data will be available as supplementary file (Data Availability statement has also been modified accordingly)

**Comments:** 4. Is the manuscript presented in an intelligible fashion and written in standard English? PLOS ONE does not copyedit accepted manuscripts, so the language in submitted articles must be clear, correct, and unambiguous. Any typographical or grammatical errors should be corrected at revision, so please note any specific errors here.

Reviewer #1: Yes

Reviewer #2: Yes

**Response:** As per various suggestions from journal Editorials and reviewers, required changes have been incorporated.

**Comments: 5. Review Comments to the Author**
Please use the space provided to explain your answers to the questions above. You may also include additional comments for the author, including concerns about dual publication, research ethics, or publication ethics. (Please upload your review as an attachment if it exceeds 20,000 characters)

**Reviewer #1:** Introduction may be shortened. Rationale for conducting this validation study should be highlighted.

**Response**: Necessary changes have been incorporated in the revised manuscript as suggested
**Comments:** Sample size needs to be recalculated.

**Response**: Sample size has been recalculated as per the suggestion.

**Comments:** Calculation of sensitivity and specificity for differentiating SS with AS should be rechecked.

**Response**: As per the suggestions, we have re-checked and necessary modifications have been incorporated (in revised table-3)

**Comments:** In tables percentage of AA, AS and SS should be given.

**Response**: Revised

**Comments:** approximate cost per test should be given.

**Response**: Mentioned in the discussion part.

**Comments:** Limit of detection of HbS and HbA should be tested.

**Response**: Limit of detection for SICKLECHECK has been analyzed and incorporated in the revised manuscript both in methods, results and discussion parts

**Comments:** How SICKLECHECK is comparable to other RDTs available in the market should be discussed in detail.

**Response**: A comparative analysis of other RDT kits have been discussed in details in the revised manuscript

**Comments**

Reviewer #2: The manuscript describes an Evaluation of a point-of-care rapid diagnostic test kit (SICKLECHECKTM) for screening of sickle cell disorders, comparing it with HPLC for diagnosis. The manuscript is prospective and descriptive comparing the new screening test against the commonly used gold standard. It is well written; however, some points need to be addressed.
General suggestion:
**Comments:** 1. Use full forms of all abbreviations at first use- both in tables and text.
**Response**: Necessary modifications have been incorporated

**Comments:** Abstract:
1. .Introduction
1. Instead of mentioning various states and disadvantaged groups- keep it general as Central India/ tribal belt, for a general audience of the journal.

**Response**: Modified as per the suggestions
2. General discussion on SCD can be omitted.

**Response**: Modified as per the suggestions

**Comments:** Methods
1. Blinding of the hospital name is advisable when describing the hospital setting.

**Response**: Modified as per the suggestions

**Comments:**2. Please mention.
a. Inclusion and exclusion criteria in more detail.

**Response**: Modified as per the suggestions

**Comments:** b. Were all consecutive patients enrolled, age group?

**Response**: All the consecutive patients were enrolled for this study. Age range: more than 6 months of age. Already mentioned in the manuscript in different sections.

**Comments:**c. Any specific criteria for screening or are all patients considered for testing?

**Response**: All patients were enrolled for this study.

**Comments:**d. What sample was used in the current study- venous blood or finger prick?

**Response**: Venous blood. However both venous blood and finger prick can be used for testing (mentioned in the manuscript)

**Comments:**e. Any time gap recommended from the last blood transfusion before testing the individuals?

**Response**: As all the ‘SS’ cases with blood transfusion were resulted with ‘SS’ pattern. No time gap is fixed for blood transfusion. But recommended to test after 3 months of blood transfusion for clear results.

**Comments**: 3. Whether the kit results are influenced by blood transfusions?

**Response**: No. All the 4 ‘SS’ cases with blood transfusion were resulted with ‘SS’ pattern. However we have recommended to test after 3 months of blood transfusion for clear results.

**Comments:** 4. Did the authors perform genetic testing for discordant results for confirmation of diagnosis? This will be beneficial for overall study findings.
**Response**: No, we have only compared with the HPLC (variant-II) results

**Comments:** 5. Funding agency details can be omitted from the methods section.
**Response**: Modified as per the suggestions

**Comments:** Results: 1. Repetition of results in text and tables can be minimized.
**Response**: Modified as per the suggestions

**Comments:** 2. It will be helpful, if authors can provide the median time to diagnosis of SCD, in their settings or previously published data in their region.
**Response**: Modified as per the suggestions and mentioned in the discussion section.

**Discussion**
**Comments:** 1. Comparative analysis with other POC test kits can be added to text (in short) and detail in a table.
**Response**: Modified as per the suggestions and mentioned in the discussion section.
Tables
**Comments:** 1. Tables 1 and 2 appear to convey the same information. Only Table 2 be retained.

**Response**: Modified as per the suggestions

**Comments:** 2. For table 5, it will be better if the final diagnosis of the patients is also listed, including the reason for low Hb.

**Response**: As we have collected the samples from the testing units, the details of final diagnosis is unavailable.

Figure:
**Comments:** 1. Figures 1 and 3 convey similar findings, either of them can be omitted.

**Response**: Modified as per the suggestions

**Comments:** 2. For a scientific journal, utility of figure 2 is also minimal- can be omitted.

**Response**: Omitted as per the suggestions

**Comments:** 3. Figure 4 and table 4 are giving same information. Figure 4 can be omitted.

**Response**: The information in Figure 4 (now figure 2) and table 4 (now table 3) are different. Figure 2 is for the sensitivity and specificity calculated for the presence and absence of Hb S (from 400 cases); while Table 3 is for genotypes pattern i.e. AA, AS and SS (359 cases).

**Comments:** While revising your submission, please upload your figure files to the Preflight Analysis and Conversion Engine (PACE) digital diagnostic tool, <https://pacev2.apexcovantage.com/>. PACE helps ensure that figures meet PLOS requirements. To use PACE, you must first register as a user. Registration is free. Then, login and navigate to the UPLOAD tab, where you will find detailed instructions on how to use the tool. If you encounter any issues or have any questions when using PACE, please email PLOS at <a href="mailto:figures@plos.org">figures@plos.org. Please note that Supporting Information files do not need this step.

**Response**: Modified accordingly as per the suggestions

**Comments: (in track changes of pdf file)**

In the above para it is mentioned that HbS was present in 164 cases in HPLC and 163 cases in Sicklecheck. Now authors calculate the sensitivity using 155 samples only. It is contradictory.

**Response**: Initially the analyses were made for the presence and absence of Hb S in all the recruited 400 cases. Further, as the SICKLECHECK^TM^ RDT kit is designed for the diagnosis of SCD only, we have calculated the sensitivity and specificity from 359 cases including 204 cases of the ‘AA’ pattern, 107 cases of the ‘AS’ pattern and 48 cases of the ‘SS’ pattern resulted in HPLC. Both the analyses have been mentioned in the methods section.
