# Supplementary material for: Evaluation of a point-of-care rapid diagnostic test kit (SICKLECHECK) for screening of sickle cell diseases
Source: PLoS One. 2024 Aug 16;19(8):e0309045. doi: 10.1371/journal.pone.0309045 (PMC11329129; doi:10.1371/journal.pone.0309045)
Supplement: Attachment — Submitted filename: RESPONSE TO REVIEWERS.docx [file pone.0309045.s005.docx]

RESPONSE TO REVIEWERS

PONE-D-24-04629R1

Evaluation of a point-of-care rapid diagnostic test kit (SICKLECHECKTM) for screening of sickle cell diseases

PLOS ONE


**Review Comments to the Author**

Reviewer-1

**Comments :** In discussion, it is written that HPLC took 24-48 hours for results which is not correct. HPLC required only 6.5 minutes per sample.

**Response:** It was written from samples collections to result dispatched. Accordingly, the sentence has been corrected in the revised manuscript.

**Comments:** "the double heterozygous states of HbS and β thalassemia diagnosed by HPLC" HPLC cannot differentiate homozygous sickle cell and S-Beta thalassemia.

**Response:** We agree with the reviewer’s comment. The double heterozygous states of HbS and β thalassemia has been diagnosed on the basis of both HbA2 level as well as red cell pictures and parents study where required.

Reviewer 2

**Comments:**  Authors have addressed most of the reviewer's comments. I feel discussion and introduction section can be shortened further, minimize general information on the disease.

**Response:** Necessary corrections have been incorporated in the revised manuscript.
